# 87 An Augmented Reality Burn Management Application to Guide Care in Austere Environments

**DOI:** 10.1093/jbcr/irad045.060

**Published:** 2023-05-15

**Authors:** Maria Serio-Melvin, Nicole Caldwell, David Luellen, Angela Samosorn, Craig Fenrich, Allison McGlasson, Patricia Colston, Laura Scott, Jose Salinas, Sena Veazey

**Affiliations:** U.S. Army Institute of Surgical Research, Ft. Sam Houston, Texas; U.S. Army Institute of Surgical Research, Ft Sam Houston, Texas; USAISR, Ft Sam Houston, Texas; USAISR, Ft. Sam Houston, Texas; USAISR, Ft Sam Houston, Texas; USAISR, FT. Sam Houston, Texas; USAISR, San Antonio, Texas; CDID, Ft. Sam Houston, Texas; USAISR, Ft Sam Houston, Texas; USAISR, San Antonio, Texas; U.S. Army Institute of Surgical Research, Ft. Sam Houston, Texas; U.S. Army Institute of Surgical Research, Ft Sam Houston, Texas; USAISR, Ft Sam Houston, Texas; USAISR, Ft. Sam Houston, Texas; USAISR, Ft Sam Houston, Texas; USAISR, FT. Sam Houston, Texas; USAISR, San Antonio, Texas; CDID, Ft. Sam Houston, Texas; USAISR, Ft Sam Houston, Texas; USAISR, San Antonio, Texas; U.S. Army Institute of Surgical Research, Ft. Sam Houston, Texas; U.S. Army Institute of Surgical Research, Ft Sam Houston, Texas; USAISR, Ft Sam Houston, Texas; USAISR, Ft. Sam Houston, Texas; USAISR, Ft Sam Houston, Texas; USAISR, FT. Sam Houston, Texas; USAISR, San Antonio, Texas; CDID, Ft. Sam Houston, Texas; USAISR, Ft Sam Houston, Texas; USAISR, San Antonio, Texas; U.S. Army Institute of Surgical Research, Ft. Sam Houston, Texas; U.S. Army Institute of Surgical Research, Ft Sam Houston, Texas; USAISR, Ft Sam Houston, Texas; USAISR, Ft. Sam Houston, Texas; USAISR, Ft Sam Houston, Texas; USAISR, FT. Sam Houston, Texas; USAISR, San Antonio, Texas; CDID, Ft. Sam Houston, Texas; USAISR, Ft Sam Houston, Texas; USAISR, San Antonio, Texas; U.S. Army Institute of Surgical Research, Ft. Sam Houston, Texas; U.S. Army Institute of Surgical Research, Ft Sam Houston, Texas; USAISR, Ft Sam Houston, Texas; USAISR, Ft. Sam Houston, Texas; USAISR, Ft Sam Houston, Texas; USAISR, FT. Sam Houston, Texas; USAISR, San Antonio, Texas; CDID, Ft. Sam Houston, Texas; USAISR, Ft Sam Houston, Texas; USAISR, San Antonio, Texas; U.S. Army Institute of Surgical Research, Ft. Sam Houston, Texas; U.S. Army Institute of Surgical Research, Ft Sam Houston, Texas; USAISR, Ft Sam Houston, Texas; USAISR, Ft. Sam Houston, Texas; USAISR, Ft Sam Houston, Texas; USAISR, FT. Sam Houston, Texas; USAISR, San Antonio, Texas; CDID, Ft. Sam Houston, Texas; USAISR, Ft Sam Houston, Texas; USAISR, San Antonio, Texas; U.S. Army Institute of Surgical Research, Ft. Sam Houston, Texas; U.S. Army Institute of Surgical Research, Ft Sam Houston, Texas; USAISR, Ft Sam Houston, Texas; USAISR, Ft. Sam Houston, Texas; USAISR, Ft Sam Houston, Texas; USAISR, FT. Sam Houston, Texas; USAISR, San Antonio, Texas; CDID, Ft. Sam Houston, Texas; USAISR, Ft Sam Houston, Texas; USAISR, San Antonio, Texas; U.S. Army Institute of Surgical Research, Ft. Sam Houston, Texas; U.S. Army Institute of Surgical Research, Ft Sam Houston, Texas; USAISR, Ft Sam Houston, Texas; USAISR, Ft. Sam Houston, Texas; USAISR, Ft Sam Houston, Texas; USAISR, FT. Sam Houston, Texas; USAISR, San Antonio, Texas; CDID, Ft. Sam Houston, Texas; USAISR, Ft Sam Houston, Texas; USAISR, San Antonio, Texas; U.S. Army Institute of Surgical Research, Ft. Sam Houston, Texas; U.S. Army Institute of Surgical Research, Ft Sam Houston, Texas; USAISR, Ft Sam Houston, Texas; USAISR, Ft. Sam Houston, Texas; USAISR, Ft Sam Houston, Texas; USAISR, FT. Sam Houston, Texas; USAISR, San Antonio, Texas; CDID, Ft. Sam Houston, Texas; USAISR, Ft Sam Houston, Texas; USAISR, San Antonio, Texas; U.S. Army Institute of Surgical Research, Ft. Sam Houston, Texas; U.S. Army Institute of Surgical Research, Ft Sam Houston, Texas; USAISR, Ft Sam Houston, Texas; USAISR, Ft. Sam Houston, Texas; USAISR, Ft Sam Houston, Texas; USAISR, FT. Sam Houston, Texas; USAISR, San Antonio, Texas; CDID, Ft. Sam Houston, Texas; USAISR, Ft Sam Houston, Texas; USAISR, San Antonio, Texas

## Abstract

**Introduction:**

Novel medical technologies are necessary to support virtual health in low bandwidth environments. When tele-mentoring is not possible, advanced clinical decision support tools can deliver essential medical knowledge to assist novice clinicians. These tools can fill knowledge gaps in specialty fields, like burn. We developed the Augmented Reality Burn Assist Manager (ARBAM), a novel, comprehensive augmented reality (AR)-based burn management platform to address key tasks in burn care.

**Methods:**

A randomized 2x2 cross-over design was used to investigate if ARBAM could enhance a user’s ability to care for a simulated burn patient as compared to standard clinical practice guidelines (CPGs). We developed the software for use on a mixed reality, head-mounted device. Participants were clinicians with little to no burn care experience, and were asked to complete 4 key tasks, twice each, using either paper copies of the Joint Trauma System’s CPGs and supplemental worksheets first (Paper) or our AR technology. Tasks included calculating burn size (TBSA), performing medication dosage calculations (MC), managing a burn fluid resuscitation (FR), and performing a simulated escharotomy (EC). All tasks were evaluated for completion time and accuracy. Cross-over analysis of treatment effects used a two-sided, two-sample t-test or Wilcoxon Rank-Sum test as appropriate.

**Results:**

Eleven participants were randomized: 5 started with Paper and 6 started with AR. Time to perform FR was statistically different (p< 0.05) with AR taking less time than Paper (4.8 vs. 7.0 mins, respectively). FR clinical performance was also better in the AR group with a mean accuracy score of 99% vs. 52% for Paper (p< 0.001). EC completion times were not clinically or statistically different. However, EC accuracy was 93% for AR compared to 55% for Paper (p< 0.001). Although not statistically different, MC performance accuracy was considerably better for AR (91%) than for Paper (66%). No differences between groups were observed for TBSA or MC completion times nor TBSA accuracy.

**Conclusions:**

Compared to traditional tools, AR may enhance a clinician's ability to complete key tasks in the initial management of a burn patient. This technology may be helpful during disasters when tele-mentoring is not available. Limitations of this study include a small sample size, limited user training on the AR device, and potential carry-over effects. Therefore, generalizations cannot be made about wide-scale usage. Nonetheless, this study showed that AR may be helpful in managing lifesaving burn tasks.

**Applicability of Research to Practice:**

ARBAM may enhance decision making by novice clinicians when caring for burn patients. Our data demonstrate the potential for AR-based support tools to improve burn care delivery and reduce error rates for novice clinicians, which could lead to reductions in morbidity and mortality. Future research is needed to investigate how AR technology can be used for other lifesaving interventions.

